# Comparison of complications secondary to cardiopulmonary resuscitation between out-of-hospital cardiac arrest and in-hospital cardiac arrest

**DOI:** 10.1186/cc14499

**Published:** 2015-03-16

**Authors:** M Seung, Y Park

**Affiliations:** 1 Yonsei University Severance Hospital/Yonsei University College of Medicine, Seoul, South Korea

## Introduction

Chest compression during cardiopulmonary resuscitation (CPR) could bring out unintended complications which are mainly composed of chest injuries. The aim of this study was to assess whether there was a significant difference in the complications of CPR between out-of-hospital cardiac arrest (OHCA) and in-hospital cardiac arrest (IHCA) survivors using multidetector computed tomography (MDCT).

## Methods

We performed a retrospective cohort study in the emergency departments of two academic tertiary care centres from January 2009 to May 2014. We enrolled both OHCA and IHCA patients who underwent successful CPR. The enrolled patients had undergone a chest CT within 48 hours after ROSC. We evaluated the MDCT findings of the CPR-related chest injuries and compared complications between OHCA and IHCA patients.

## Results

**Figure 1:**
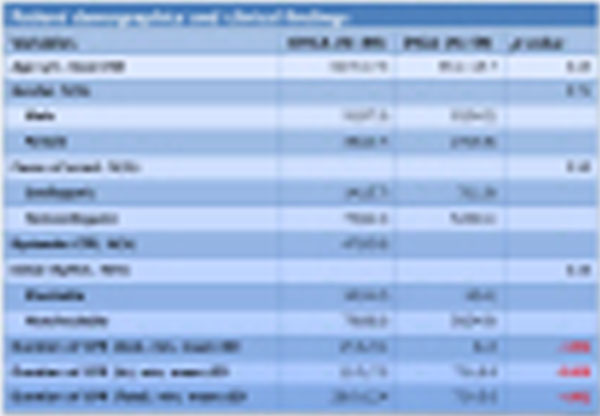
**Patient demographics and clinical findings**.

**Figure 2:**
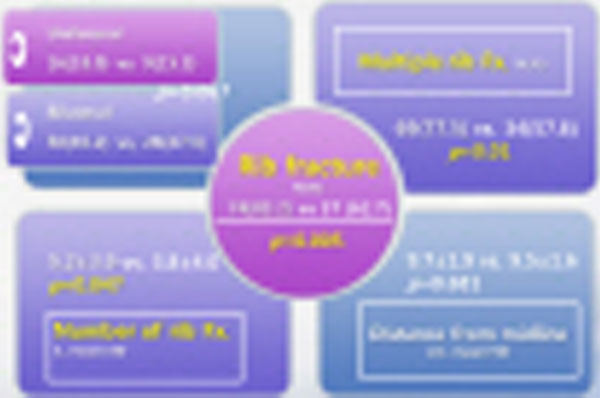
**Comparison complications of CPR between the OHCA group and the IHCA group**.

A total of 148 patients were finally enrolled in this study, OHCA were 89 (60.1%) and IHCA were 59 (39.8%). The mean CPR time, both in-hospital and total, was longer in OHCA survivors. Rib fractures were detected more in OHCA survivors. Frequency of multiple rib fractures was higher in OHCA survivors. Frequency of sternum fractures was higher in OHCA survivors, showing no significant difference. In lung injuries, lung contusion and pneumothorax account for the large part, and OHCA survivors had higher incidence in both complications but statistically insignificant. Major complications occurred in eight cases in OHCA survivors and three cases in IHCA survivors during the study period. After adjusting for the time factor in multiple logistic regression analysis, rib fractures and multiple rib fractures became statistically significant in OHCA survivors. See Figures 1 and 2.**[AQ1]**

## Conclusion

Frequency of rib fractures and multiple rib fractures were higher in OHCA survivors. Further investigation is needed into the relation between the location of CPR and the CPR-related injuries, efforts to reduce the complications after CPR.

## References

[B1] Kim EY Yang HJ Sung YM Multidetector CT findings of skeletal chest injuries secondary to cardiopulmonary resuscitation Resuscitation 2011 82 1285 8 21705131 10.1016/j.resuscitation.2011.05.023

[B2] Kim MJ Park YS Kim SW Chest injury following cardiopulmonary resuscitation: a prospective computed tomography evaluation Resuscitation 2013 84 361 4 22819881 10.1016/j.resuscitation.2012.07.011

